# Characteristics of facial expression recognition ability in patients with Lewy body disease

**DOI:** 10.1186/s12199-018-0723-2

**Published:** 2018-07-18

**Authors:** Yuriko Kojima, Tomohiro Kumagai, Tomoo Hidaka, Takeyasu Kakamu, Shota Endo, Yayoi Mori, Tadashi Tsukamoto, Takashi Sakamoto, Miho Murata, Takehito Hayakawa, Tetsuhito Fukushima

**Affiliations:** 1 Department of Hygiene and Preventive Medicine, Fukushima Medical University School of Medicine, Hikarigaoka 1, Fukushima, 960-1295 Japan; 2 Department of Neurology, National Center of Neurology and Psychiatry, Kogawahigashi-cho 4-1-1, Kodaira, Tokyo 187-8551 Japan; 3 Research Center for Social Studies of Health and Community, Ritsumeikan University, Tojiinkita-machi 56-1, Kita-ku, Kyoto, 603-8577 Japan

**Keywords:** Facial expression recognition, Basic facial expressions, Aging, Parkinson’s disease, Lewy body disease, Dementia

## Abstract

**Background:**

The facial expression of medical staff has been known to greatly affect the psychological state of patients, making them feel uneasy or conversely, cheering them up. By clarifying the characteristics of facial expression recognition ability in patients with Lewy body disease, the aim of this study is to examine points to facilitate smooth communication between caregivers and patients with the disease whose cognitive function has deteriorated.

**Methods:**

During the period from March 2016 to July 2017, we examined the characteristics of recognition of the six facial expressions of “happiness,” “sadness,” “fear,” “anger,” “surprise,” and “disgust” for 107 people aged 60 years or more, both outpatient and inpatient, who hospital specialists had diagnosed with Lewy body diseases of Parkinson’s disease, Parkinson’s disease with dementia, and dementia with Lewy bodies. Based on facial expression recognition test results, we classified them by cluster analysis and clarified features of each type.

**Results:**

In patients with Lewy body disease, happiness was kept unaffected by aging, age of onset, duration of the disease, cognitive function, and apathy; however, recognizing the facial expression of fear was difficult. In addition, due to aging, cognitive decline, and apathy, the facial expression recognition ability for sadness and anger decreased. In particular, cognitive decline reduced recognition of all of the facial expressions except for happiness. The test accuracy rates were classified into three types using the cluster analysis: “stable type,” “mixed type,” and “reduced type”. In the “reduced type”, the overall facial recognition ability declined except happiness, and in the mixed type, recognition ability of anger particularly declined.

**Conclusion:**

There were several facial expressions that the Lewy body disease patients were unable to accurately identify. Caregivers are recommended to make an effort to compensate for such situations with language or body contact, etc., as a way to convey correct feeling to the patients of each type.

## Background

The six facial expressions of “happiness,” “sadness,” “fear,” “anger,” “surprise,” and “disgust” are agreed to be basic expressions regardless of race, language, and culture [1, 2]. In previous studies, facial expression recognition tests have been conducted with a method presenting stimuli based on the classification of these six basic expressions. As a result, it turned out that there are diseases that hinder recognition of facial expressions. According to Ruffman et al.’s meta-analysis of previous research comparing elderly and young people’s facial expression recognition [3], the average accuracy rate on the facial expression recognition test for young people compared with the elderly group was happiness (98%) > sadness (89%) > surprise (87%) > disgust (81%) > fear (79%), so even without the effect of aging, there was a difference for young people in the degree of difficulty of recognition for each facial expression.

In psychiatry and cognitive neurology, it has been reported that autistic children, people with schizophrenia, alexithymic individuals, and traumatic brain injury patients have poor facial expression recognition ability [4–7]. Parkinson’s disease (PD) patients showed a decline in recognition ability of fear and disgust; patients with myotonic dystrophy type I had decreased recognition of anger and disgust; and those with Huntington’s disease showed a decline in recognition ability of disgust [8–11].

There has also been research with respect to facial expression recognition in the elderly. Calder et al. found that although there was a decline in recognition ability of fear and anger, there was no decline in recognition ability of disgust [12]. The results of McDowell et al. indicate that there was a decline in recognition ability of neutral and negative facial expressions, but there was no decline in recognition ability of happiness [13]. Phillips et al. found a decline in recognition ability of anger and sadness [14]. Although the results of these previous studies are not completely consistent, they do show that there is not a decline in recognition ability of all facial expressions due to the influence of aging in elderly people; rather, recognition of fear, anger, and sadness is reduced, while the ability to recognize happiness does not decrease.

As an extension of the elderly study, Maki et al. used results of perceptual matching challenges of patients with Alzheimer’s disease to compare with results of healthy elderly people and young adults and found no significant difference with the healthy elderly [15]. Results of this study suggest that happiness, which is a positive facial expression, does not show a decline due to age, but any consistent trends about facial expression recognition ability of elderly people with dementia are not clear.

After the onset of PD, the number of those with dementia increases with the passage of years; after 20 years, it has been reported that 83% of patients will have dementia [16]. Various types of PD-related dementia include Parkinson’s disease with dementia (PDD) [17], in which dementia develops several years after the onset of PD, as distinguished from dementia with Lewy bodies (DLB) [18], in which dementia develops within 1 year after the appearance of Parkinsonism. PD, PDD, and DLB have come to be collectively referred to as Lewy body disease [19] because proteins called alpha-synuclein accumulate and form aggregates called Lewy bodies which can be seen in nerve cells.

This study focused on Lewy body disease and revealed the characteristics of facial recognition function in Lewy body disease patients which has not been clarified, with classification made using cluster analysis. Based on the results of this analysis, we examined points to be considered for facilitating smooth communication between caregivers and patients with impaired cognitive function.

## Methods

### Subjects

This study was conducted from March 2016 to July 2017 by specialists from the National Center of Neurology and Psychiatry Hospital, Japan, with 107 subjects (outpatient and inpatient) 60 years of age or older diagnosed with Lewy body disease (PD [20], PDD [17], and DLB [21]). Subjects were judged by a specialist to be able to communicate intentionally, and the breakdown by diagnosis was PD (80 people), PDD (8 people), and DLB (19 people).

### Measures

Survey items included basic attributes, a cognitive function test, face-to-face questionnaires (depression scale, anxiety scale, and motivation reduction scale), and a test for facial expression recognition by facial stimuli. Electronic medical record data was used for the basic attributes of age, sex, onset age, and duration of disease.

#### Tests for basic attributes

For the cognitive function test, the Mini-Mental State Examination (MMSE) was used [22]. A score of 24 points or more was determined as non-dementia, and a score of 23 points or less was defined as dementia; for the depression scale, subjects completed the Geriatric Depression Scale short-version (GDS-15), a simple 15-question test with two choices, “yes” or “no,” which has shown relatively high sensitivity [23] as an evaluation criterion for depressive symptoms of PD [24]. A score of 6 or more suggests depression. The State-Trait Anxiety Inventory (STAI) was used as an anxiety scale [25], making it possible to measure both state anxiety and trait anxiety with 20 questions each. Fifty-five points or more points to high anxiety; the Apathy Scale was used to measure reduction in motivation [26], and a score of 16 points or more has been determined to suggest apathy.

#### Test for facial expression recognition

Regarding the test for facial expression recognition based on facial stimulation (the facial expression recognition test), this study used 42 facial pictures classified by Ekman et al. [27] as the six basic expressions (happiness, sadness, fear, anger, surprise, and disgust) reported to be commonly recognized in all countries [1, 2], in order to investigate how subjects understand others’ feelings. Subjects were randomly presented with facial pictures one by one showing one of the six facial expressions and instructed to decide which of the six basic facial expressions that picture was expressing. No particular time limit was set, and the photograph was placed so as to be in the field of view of the subject the whole time.

### Statistical analysis

The mean and standard deviation were found for the basic attributes of the subject: age, onset age, and duration of disease; MMSE score; GDS-15 score; anxiety score, state anxiety (STAI-1) and trait anxiety (STAI-2); and Apathy Scale score. Median and 25–75 percentiles were calculated for the accuracy of recognition test.

Subjects were classified into three groups based on the diagnosis (PD, PDD, and DLB). Then, the accuracy rates of the facial expression recognition test were calculated by the classification. The Mann-Whitney *U* test was used for comparisons of the facial expression recognition accuracy rate, and the degree of relevance between the basic attribute items of the subject and the facial expression recognition accuracy rate was evaluated by Spearman rank correlation. Subsequently, hierarchical cluster analysis (Ward method) was performed to identify the characteristics of the subject from the combination patterns of the six facial expression recognition test accuracy rates.

To describe the characteristics of each cluster, we compared the age, onset age, sex, MMSE score, Apathy Scale score, and diagnosis of the patients. We categorized the cutoff values of MMSE and Apathy Scale scores as 24 and 16, respectively. For age and onset age, we used one-way analysis of variance (ANOVA) test and the Bonferroni method for multiple comparisons. Chi-square test and residual analysis were used for sex, MMSE score, Apathy Scale score, and diagnosis. The cells were considered to have significantly more subjects than expected when the adjusted standardized residual values were greater than 1.96, whereas the cells were considered to have significantly fewer subjects than expected when the values were lower than − 1.96.

All statistical analyses were performed by using statistical software SPSS Statistics version 24 (IBM Corp., Armonk, NY, USA). The significance level was set at 5%.

### Ethics

The objectives and content of this research, management of personal information, and anonymity of answers were all explained in writing, and written consent was received. This study was approved by the Fukushima Medical University Ethics Committee (approval number 2426) and the ethics committee of the National Center of Neurology and Psychiatry Hospital (approval number A2015-82).

## Results

A comparison of facial expression recognition test accuracy rates is shown in Table 1. There was no significant difference between PD + PDD and DLB in their facial expression recognition test accuracy rate. It was determined that there were no vision-related effects, visual hallucinations of DLB.

**Table 1:** Comparison of diagnosis breakdown and facial expression recognition test accuracy

	PD + PDD (*n* = 80 + 8)			DLB (*n* = 19)			*P* value
	Percentile			Percentile			
	25	50	75	25	50	75	
Happiness	85.7	100.0	100.0	100.0	100.0	100.0	0.193
Sadness	62.5	75.0	87.5	62.5	75.0	87.5	0.350
Fear	16.6	16.7	33.3	0.0	16.7	33.3	0.070
Anger	28.6	57.1	71.4	28.6	42.9	57.1	0.170
Surprise	62.5	75.0	87.5	50.0	62.5	75.0	0.055
Disgust	16.7	33.3	66.7	16.7	16.7	50.0	0.100

*P* value: Mann-Whitney *U* test

Mean, median, and interquartile range of basic attribute items of subjects are shown in Table 2. The overall average age was 74.7 years of age (minimum 60 years, maximum 94 years), and the average age of males was 73.5 years while the average age of females was 75.9 years old. The overall average age of onset was 67.9 years of age (minimum 48 years, maximum 88 years) with an average age of onset for males at 66.7 years and for females 69.0 years. The overall average duration of the disease was 6.8 years (minimum less than 1 year, maximum 19 years), with an average duration of disease in males as 6.9 years and females 6.8 years.

**Table 2:** Basic attributes of subjects

	Total (*n* = 107)	Male (*n* = 52)	Female (*n* = 55)	PD + PDD (*n* = 80 + 8)	DLB (*n* = 19)
	Mean ± SD	Mean ± SD	Mean ± SD	Mean ± SD	Mean ± SD
Age (year)	74.7 ± 7.8	73.5 ± 6.6	75.9 ± 8.6	73.5 ± 7.4	80.7 ± 7.0
Age of onset (year)	67.9 ± 9.1	66.7 ± 8.1	69.0 ± 9.9	66.0 ± 8.5	76.7 ± 6.8
Duration of disease (year)	6.8 ± 4.9	6.9 ± 4.3	6.8 ± 5.4	7.5 ± 5.0	4.0 ± 3.2
Male (%)				44 (50.0)	8 (42.1)
MMSE (cognitive function)	24.8 ± 4.3	25.1 ± 4.3	24.6 ± 4.3	25.5 ± 4.1	21.8 ± 3.5
GDS-15 (depression)	4.9 ± 3.1	4.7 ± 3.1	5.1 ± 3.2	4.8 ± 3.3	5.3 ± 2.4
STAI-1 (state anxiety)	42.9 ± 7.9	42.1 ± 8.1	43.7 ± 7.7	42.9 ± 8.3	42.9 ± 6.2
STAI-2 (trait anxiety)	42.8 ± 8.4	42.1 ± 8.1	43.4 ± 8.8	43.0 ± 8.8	41.7 ± 6.0
Apathy (motivation reduction)	13.6 ± 5.6	13.8 ± 5.9	13.5 ± 5.3	13.6 ± 5.8	13.8 ± 4.8

*SD* standard deviation

The mean and median values of MMSE, GDS-15, STAI, and Apathy Scale were within the range of normal levels for total, male, female, PD, and DLB categories. The variation seen in the interquartile range was also roughly constant for the total, male, and female values.

Table 3 shows a comparison of facial expression recognition test accuracy by gender. With the facial expression of happiness, the results were significantly higher for women than for men. Outside of happiness, a statistically significant difference could not be seen with sadness, fear, anger, surprise, and disgust. A ranking of the facial expression recognition test accuracy rates showed the same trend, and in both men and women, the rankings were happiness > sadness and surprise > anger > disgust > fear.

**Table 3:** Comparison of facial expression recognition test accuracy by gender

	Male (*n* = 52)			Female (*n* = 55)			*P* value
	Percentile			Percentile			
	25	50	75	25	50	75	
Happiness	85.7	100.0	100.0	100.0	100.0	100.0	0.040*
Sadness	62.5	75.0	87.5	75.0	75.0	100.0	0.224
Fear	4.2	16.7	33.3	16.2	16.7	33.3	0.596
Anger	28.6	57.1	71.4	28.6	57.1	71.4	0.830
Surprise	62.5	75.0	87.5	62.5	75.0	87.5	0.944
Disgust	16.7	33.3	50.0	16.7	50.0	66.7	0.117

*P* value: Mann-Whitney *U* test; *statistical significance

The relevance between subjects’ basic attribute items and facial expression recognition test accuracy rates was evaluated by Spearman’s rank correlation with results shown in Table 4. Of the six facial expressions, except for happiness and fear, there were significant positive correlations between the facial expressions of sadness and surprise, sadness and disgust, anger and surprise, and surprise and disgust. There were significant negative correlations between age and the three facial expressions of sadness, anger, and surprise. There were significant negative correlations between the age of onset and anger and surprise. Other than happiness, MMSE had significant positive correlations with facial expression recognition of sadness, fear, anger, surprise, and disgust. There were significant negative correlations between Apathy Scale score and the two facial expressions of sadness and anger.

**Table 4:** Spearman’s rank correlation of facial expression recognition test accuracy rate and basic attribute items

	Age	Onset age	Disease duration	MMSE	GDS-15	STAI-1	STAI-2	Apathy	Happiness	Sadness	Fear	Anger	Surprise	Disgust
Age	–	0.835**	0.033	− 0.481**	0.123	0.110	0.033	0.200*	− 0.136	− 0.222*	− 0.038	− 0.244*	− 0.261**	− 0.074
Onset age		–	− 0.479**	− 0.370 **	0.006	− 0.004	− 0.053	0.089	− 0.079	− 0.117	− 0.002	− 0.227*	− 0.208 *	− 0.077
Disease duration			–	− 0.110	0.135	0.136	0.125	0.150	− 0.103	−0.105	− 0.084	0.027	− 0.019	− 0.027
MMSE				–	− 0.229 *	− 0.216*	− 0.128	− 0.298 **	0.072	0.238*	0.203*	0.295**	0.273**	0.341**
GDS-15					–	0.524**	0.606 **	0.437 **	0.049	− 0.186	− 0.123	0.120	− 0.057	0.018
STAI-1						–	0.678 **	0.441 **	0.064	0.184	− 0.027	− 0.096	− 0.085	− 0.156
STAI-2							–	0.327 **	0.100	− 0.104	0.038	0.095	0.049	0.027
Apathy								–	0.027	− 0.215*	− 0.054	− 0.194*	− 0.145	− 0.097
Happiness									–	0.104	0.041	0.128	0.016	0.110
Sadness										–	0.043	0.133	0.298**	0.232*
Fear											–	− 0.021	− 0.009	0.160
Anger												–	0.235 *	0.179
Surprise													–	0.236 *

*P < 0.05, **P < 0.01

**Fig. 1:**
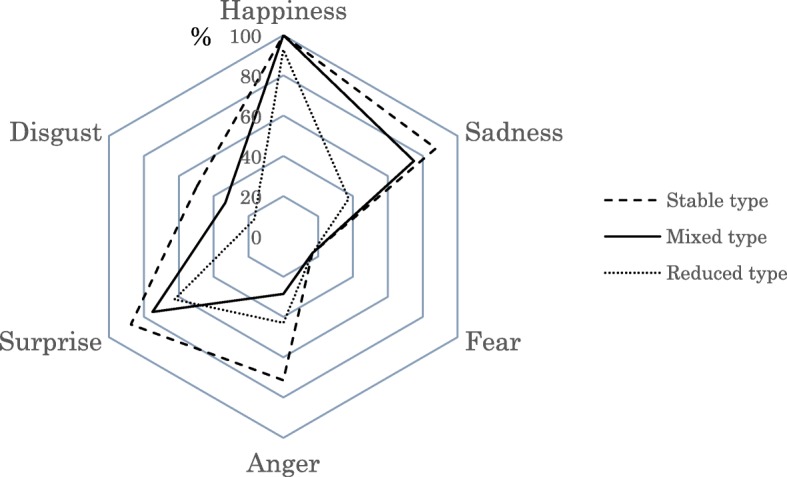
Facial expression recognition accuracy rate by type

From the combination patterns of the six facial expression recognition test accuracy rates, hierarchical cluster analysis was conducted, and the facial expression recognition test accuracy rates (median) were classified into three types (Fig. 1). The cluster with a facial expression recognition test accuracy rate higher than the other two clusters was labeled the “stable type” (45 subjects). The ranking of each facial expression recognition test accuracy rate of the stable type was happiness (100%) > sadness (87.5%) and surprise (87.5%) > anger (71.4%) > disgust (49.6%) > fear (16.7%).

The cluster with an overall accuracy rate lower than the other two clusters was labeled the “reduced type” (22 subjects). The ranking of each facial expression recognition test accuracy rate of the reduced type was happiness (92.9%) > surprise (62.5%) > anger (42.9%) > sadness (37.5%) > disgust (16.7%) and fear (16.7%). The reduced type was characterized by inability to identify sadness and disgust in the facial expression recognition test.

The cluster with accuracy rates between the stable type and the reduced type was labeled the “mixed type” (40 subjects). The ranking of each facial expression recognition test accuracy rate of the mixed type was happiness (100%) > sadness (75.0%) and surprise (75.0%) > disgust (33.3%) > anger (28.6%) > fear (16.7%).

Happiness and fear had the same accuracy rates when comparing the facial expression recognition test accuracy rates of the mixed type and stable type, and the mixed type was lower in sadness, surprise, disgust, and anger. In particular, a major feature of the mixed type was that the accuracy rate of anger was less than that of the reduced type.

Table 5 shows basic attribute items and frequency of diagnoses that showed a relationship with facial expression recognition test accuracy rates by each type that was extracted from cluster analysis. Overall, between three clusters, statistically significant differences were observed in age, age of onset, and MMSE. Age and onset age were significantly higher in the reduced type than in the stable type. The rate of MMSE < 24 scores was significantly lower in the stable type, and reduced type showed significantly higher rate of MMSE < 24 scores. Of the subjects with DLB, 10 (52.6%) were categorized to mixed type.

**Table 5:** Comparison of attributes by type

	Age Mean ± SD	Age of onset Mean ± SD	Sex		MMSE		Apathy		Diagnosis		
			Male	Female	24 or more	Less than 24	16 or more	Less than 16	PD	PDD	DLB
Stable type (*n* = 45)	72.1 ± 7.1^a^	65.0 ± 8.6^a^	20 (38.5)	25 (45.5)	39 (56.5)^‡^	6 (15.8)^†^	13 (28.9)	32 (71.1)	38 (47.5)	3 (37.5)	4(21.1)
Mixed type (*n* = 40)	75.5 ± 8.1^a, b^	69.1 ± 9.3^a, b^	19 (36.5)	21 (38.2)	23 (33.3)	17 (44.7)	16 (40.0)	24 (60.0)	27 (33.8)	3 (37.5)	10(52.6)
Reduced type (*n* = 22)	78.7 ± 6.9^b^	71.5 ± 8.6^b^	13 (25.0)	9 (16.4)	7 (10.1)^†^	15 (39.5)^‡^	12 (54.5)	10 (45.3)	15 (18.8)	2 (25.0)	5(26.3)
*P* value	0.003*	0.013*	0.522		*< 0.001* *		0.123		0.334		

*Significant one-way ANOVA for continuous variables or chi-square test for discrete variables

^a, b^Multiple comparison by Bonferroni method

^†^Adjusted standardized residual <-1.96

^‡^Adjusted standardized residual > 1.96

*SD* standard deviation

## Discussion

This study divided the facial expression recognition ability into three types: stable type, mixed type, and reduced type, and revealed their characteristics. Reduced type was characterized by poor performance in the facial expression recognition test regarding sadness and disgust faces, and it is assumed that this result was caused by the decline of cognitive function, since there were a statistically large number of subjects with MMSE scores of less than 24 in this group. The mixed type group had a lower accuracy rate of regarding identifying angry expressions than the other two groups. Mixed type was a specific group with poor performance of recognition test only for anger face in spite of their better function of cognition than reduced type. It may be difficult for mixed type patients to understand the emotion of anger even when their caregivers show angry expressions. Thus, particular care is required when communicating the emotion of anger when treating patients who fall under this category.

With regard to happiness, the only positive expression in the six basic expressions, the facial expression recognition test accuracy remained consistently high in this research for all types (stable type, mixed type, and reduced type), revealing that facial expression recognition of happiness does not change due to age or cognitive deterioration. This supports the results of McDowell et al. [13] and Maki et al. [15]. It has also been reported that happiness (smiling) has a higher predominance of perception and identification than other facial expressions, such as disgust and sadness [28, 29]. Although the facial expression recognition test accuracy rate of happiness was high for males, results showed females judged even more accurately than men. There are sex differences in facial expression recognition, which is consistent with the finding that female facial expression recognition ability was higher than that of men [30–32]. It could be said that this reflects that generally, compared to men, women are more likely to express emotions and are more sensitive to the feelings of others.

The expression of surprise is neither positive nor negative; rather, it can be regarded as a neutral expression. Age, age of onset, and cognitive function correlated, and facial expression recognition performance showed a gradual decline due to aging and cognitive decline, which is consistent with the findings obtained from previous work by McDowell et al. [13].

In relation to the negative expressions of disgust and fear, this study included many stable type subjects without cognitive decline who scored less than a 50% accuracy rate for disgust and fear, findings which are consistent with a previous study by Kan et al. [8], in which PD patients without dementia had difficulty recognizing the facial expressions of disgust and fear. Additionally, fear was not influenced by aging, age of onset, or the duration of the disease, and the results of the facial expression recognition test were consistently low at 16.7% for the stable type, mixed type, and reduced type. With regard to disgust, although it was not affected by aging, age of onset, and the duration of the disease, there was a difference between the three types. Since compared to PD and PDD, DLB is a disease characterized by having frequent occurrences of visual hallucinations, auditory hallucinations, delirium, etc. [33], facial expression recognition tests targeting DLB patients had not been proactively conducted thus far. In the current study, we considered the possibility that DLB patients would not be able to correctly judge the facial photographs of people or to be able to participate in facial expression recognition tests due to the visual hallucinations. However, it was determined that there was no effect of visual hallucinations, suggesting that facial expression recognition tests can be performed with DLB patients henceforth.

A major characteristic of anger, a negative expression, was that its facial expression recognition test accuracy rate was significantly lower in the mixed type than the reduced type. Previous studies reported that an angry facial expression can be correctly identified more often and quicker than other expressions, since this face is a signal of direct threat [34, 35]. It has been reported that antisocial people with low empathy score lower in facial expression recognition tests that consist of negative facial expressions such as anger and fear [36, 37]. Furthermore, there is genetic modulation for facial recognition which neutral face tends to be recognized as anger [38]. Looking by diagnosis in the cluster analysis results, it can be seen that the mixed type included as many as 10 DLB subjects, which is half of the total of 19 patients with DLB. The remarkable decrease in the accuracy rate of anger seen in the mixed type may be from the possibility of a characteristic of DLB.

The current study had some limits. First, it was a cross-sectional study, and the cause and effect relationship could not be clarified. In addition, the representativeness of the sample was insufficient because the subjects of this study were recruited from single medical institution and also were small-sized. The populations of future studies should be larger than that of the current study.

## Conclusions

The patterns of facial expression recognition ability in patients with Lewy body disease were classified into three types: stable type, mixed type, and reduced type, and each category was defined. The reduced type comprised of subjects with an overall low accuracy rate for all facial expressions except happiness. The accuracy rate of anger in the mixed type was particularly low. We recommend that caregivers make an effort to compensate for such unrecognized feelings with language, body contact, etc., as a way to convey their intended feelings to the patients.

## Abbreviations

DLB: Dementia with Lewy bodies
GDS-15: Geriatric Depression Scale Short-version
MMSE: Mini-Mental State Examination
PD: Parkinson’s disease
PDD: Parkinson’s disease with dementia
